# Expression of the Memory Marker CD45RO on Helper T Cells in Macaques

**DOI:** 10.1371/journal.pone.0073969

**Published:** 2013-09-04

**Authors:** Michael Valentine, Kejing Song, Grace A. Maresh, Heather Mack, Maria Cecilia Huaman, Patricia Polacino, On Ho, Anthony Cristillo, Hye Kyung Chung, Shiu-Lok Hu, Seth H. Pincus

**Affiliations:** 1 Research Institute for Children, Children’s Hospital, New Orleans, Louisiana, United States of America; 2 Departments of Microbiology, Immunology and Parasitology, LSU Health Sciences Center, New Orleans, Louisiana, United States of America; 3 Department of Pediatrics, LSU Health Sciences Center, New Orleans, Louisiana, United States of America; 4 Washington National Primate Research Center, University of Washington, Seattle, Washington, United States of America; 5 Advanced BioScience Laboratories Inc., Rockville, Maryland, United States of America; Tulane University, United States of America

## Abstract

**Background:**

In humans it has been reported that a major site of the latent reservoir of HIV is within CD4+ T cells expressing the memory marker CD45RO, defined by the mAb UCHL1. There are conflicting reports regarding the expression of this antigen in macaques, the most relevant animal species for studying HIV pathogenesis and testing new therapies. There is now a major effort to eradicate HIV reservoirs and cure the infection. One approach is to eliminate subsets of cells housing the latent reservoir, using UCHL1 to target these cells. So that such studies may be performed in macaques, it is essential to determine expression of CD45RO.

**Methods:**

We have used immunofluorescence and flow cytometry to study cell surface expression of CD45RO on lymphocytes from PBMC, lymphoid, and GI organs of rhesus, pigtailed, and cynomolgus macaques. Both direct and indirect immunofluorescence experiments were performed.

**Findings:**

CD45RO is expressed on a subset of CD4+ lymphocytes of all pigtailed, a fraction of rhesus, and neither of the cynomolgus macaques studied. The binding of UCHL1 to macaque cells was of lower avidity than to human cells. This could be overcome by forming UCHL1 multimers. Directly conjugating fluors to UCHL1 can inhibit UCHL1 binding to macaque cells. Patterns of UCHL1 expression differ somewhat in macaques and humans, and from that of other memory markers often used in macaques.

**Conclusions:**

CD45RO, defined with mAb UCHL1, is well expressed on CD4+ cells in pigtailed macaques. Using tissues recovered from latently infected pigtailed macaques we are determining whether UCHL1, or other memory markers, can define the cellular locus of the reservoir. The low avidity of this interaction could limit the utility of UCHL1, in its conventional form, to eliminate cells in vivo and test this approach in macaque models of HIV infection.

## Introduction

CD45RO is a marker of memory T-cells [1–3]. The first monoclonal antibody (mAb) identifying human CD45RO was UCHL1 [4]. CD45 is a transmembrane protein tyrosine phosphatase, receptor type, C expressed on the cell surface of human leukocytes [5]. Several splicing variants have been described. The extracellular portion is heavily glycosylated and solely responsible for the diversity. CD45RO is the shortest isoform and has been shown to be expressed on memory but not naïve T-cells [6,7]. The exact epitope on CD45RO to which UCHL1 binds remains unclear. The UCHL1 epitope is destroyed by treatment with either neuraminidase or O-glycosidase [8]. Alanine mutation of specific O-linked glycosylation sites near the junction of exons 3 and 7 also abolished or weakened UCHL1 binding. A CD45RO expression plasmid conferred UCHL1 reactivity when transfected into human and murine cell lines, suggesting that species-specific differences in glycosylation machinery do not influence CD45RO expression.

The persistent latent reservoir of HIV, cells carrying functional provirus but not actively producing HIV, is the major obstacle to a sterilizing cure of HIV infection [9–11]. Long-lived memory CD4+ T cells represent a major cellular locus of the latent reservoir, although cells of the macrophage lineage may also contribute [12]. Elimination of these cell subsets is the most direct approach to removing the reservoir, but runs the risk of causing immunodeficiency. Vitetta and coworkers have demonstrated in HIV-infected patients effectively treated with anti-retroviral therapy (ART) that expression of CD45RO defined the latent reservoir in the peripheral blood, and further that an immunotoxin made with UCHL1 was highly effective in eliminating these cells *ex vivo* [13–15]. Macaque models of HIV infection could represent an excellent opportunity to study both the immunologic and virologic effects of such treatment [16].

The expression of CD45RO on macaque memory cells is a matter of some controversy. Firpo and coworkers originally described that this antigen could be detected in *Macaca* *nemestrina* (pigtailed macaques) with mAb UCHL1 when using indirect immunofluorescence, but not when a directly conjugated UCHL1 preparation was tested [17]. More recently, Wang et al. have indicated that the mAb UCHL1 used to detect the CD45RO isoform in humans does not react with nonhuman primates [18]. The alternative anti-CD45RO mAb from the clone OPD4 does react with ~44% of Indian-origin rhesus macaques (*M. mulatta*) [14]. Unfortunately, OPD4 is not readily available. A combination of other markers can be used for identifying memory T cells in macaques, e.g. activation markers, CD28 and CD95 [19] and chemokine receptors or homing markers such as CCR7 and CD62L [20]. But the need to use combinations of Abs, and the possibility that CD45RO and other markers may define somewhat different sets of memory cells, spurred us to define the expression of CD45RO, as defined by UCHL1, on macaque memory cells. We demonstrate that this mAb reacts with memory CD4+ cells in all pigtailed and some rhesus macaques, but that the binding is of lower avidity than that in humans.

## Materials and Methods

### Human Studies

Human blood was obtained from adult volunteers providing written consent. Blood was drawn by study nurses who provided blinded samples to investigators. The IRBs of LSU Health Sciences Center (IRB# 5030) and Children’s Hospital, New Orleans (ARC# 01-140) approved the drawing of blood from healthy donors for research purposes. Leukapheresis packs were also obtained from Research Blood Components, LLC (Brighton, MA). Cells were from a de-identified individual tested to be negative for known infections prior to leukapheresis. Commercially obtained, de-identified cells are exempt as human subjects research.

### Animal Studies

Three macaque species were studied: *Macaca* *nemestrina* (pigtailed), *M. mulatta* (rhesus) and *M. fascicularis* (cynomolgus). Most animals were infected with SHIV-162.P4, and some were uninfected. Animals were housed at the Washington National Primate Research Center (WaNPRC), the Tulane National Primate Research Center (TNPRC), and at Advanced BioScience Laboratories. All studies were performed in compliance with the National Institutes of Health “Guide for the Care and Use of Laboratory Animals”, were approved by the appropriate Institutional Animal Care and Use Committees, and all procedures were performed according to protocols pre-approved by the IACUC. Animals were under the care of a licensed veterinarian and all efforts were made to minimize animal pain and suffering, in accordance with the recommendations of the Weatherall report. Each facility was accredited by the Association for the Assessment and Accreditation of Laboratory Animal Care International and registered as a USDA Class R research facility. WaNPRC is certified by the NIH Office of Laboratory Animal Welfare (OLAW A3464-01), and experiments were performed under protocol 2370-28, approved by the University of Washington IACUC. Experiments at TNPRC (OLAW A4400-01) were performed under protocols P0092 and P0016 approved by the Tulane University IACUC.

Animal studies at Advanced BioScience Laboratories (OLAW A3467-01) were performed under IACUC-approved protocols AUP 505 and AUP 462. Animals were housed in a temperature and humidity controlled environment and were monitored by veterinary staff at least once daily. Prior to infection, animals were pair housed. Following infection, macaques were housed separately to prevent cross-transmission but were provided regular visual, aural and olfactory contact with other macaques. Waste trays and cages were cleaned and sanitized daily and biweekly, respectively. Animals were fed three meals daily of a commercial monkey chow, which was supplemented with fresh fruits and vegetables, and given access to water ad libitum. Environmental enrichment was provided by a biweekly rotation of toys, use of foraging boards, social housing where permitted and/or positive human interaction, including food treats, with veterinary and research staff. Minor manipulations, such as inoculation or phlebotomy, were performed under ketamine anesthetic (10mg/kg i.m.). Surgical procedures at ABL were performed under domitor or isoflurane anesthesia. Animals deemed moribund by the veterinary staff and those scheduled for necropsy were euthanized as follows: the animal was placed under deep anesthesia either by sodium pentobarbital or inhalation of anesthesia, followed by exsanguination, and then euthanized with an overdose of pentobarbital. The decision to euthanize was based upon any of the following criteria: presentation with AIDS defining symptoms, loss of >20% body weight, an opportunistic infection not responding to treatment, chronic diarrhea or anorexia, neurological deterioration or other signs of distress observed by the veterinary staff. Death was confirmed by auscultation for sustained cardio-pulmonary arrest.

### Antibodies and Other Reagents

Table 1 lists the Abs used in this study. The UCHL1 hybridoma, secreting Ab to CD45RO [4,21], was grown in serum free hybridoma medium (Gibco) at 37^0^ in a 5% CO_2_ humidified atmosphere. UCHL1 mAb was purified from the supernatant on a sterile column of protein A sepharose (Sigma). After elution in 0.2M glycine-HCl pH 2.8, and neutralization with 2M Tris-base (Sigma), the buffer was exchanged to phosphate buffered saline (PBS). Endotoxin level was measured by the limulus amebocyte lysate assay (Associates of Cape Cod, Inc.). To make UCHL1-streptavidin multimers, mAb (2mg/ml) was biotinylated with SulfoNHS Lc Biotin (Pierce) at a 1: 1 molar ratio in 50mM sodium bicarbonate pH 8.5 for 2hrs on ice. Biotinylated UCHL1 was separated from un-reacted SulfoNHS Lc Biotin on a Zeba desalting column (Pierce). Biotinylated UCHL1 (20µg/ml) was incubated at 4: 1 molar ratio with Streptavidin-PE (BioLegend Inc.) for 20 minutes in PBS/1% bovine serum albumin/0.01% sodium azide (PBA) prior to use. The presence of dimeric through tetrameric species was shown by fast protein liquid chromatography (data not shown). Preparations of UCHL-1 directly conjugated to fluorochromes were available commercially, and are listed in table 1, as are directly conjugated mAbs to cell surface markers other than CD45RO. These mAbs were used at the concentrations recommended by the manufacturer. The anti-CD4 mAb CD4R1 (NIH Nonhuman Primate Reagent Resource) was conjugated to Alexa-488 dye (Invitrogen) at 1: 4 molar ratio in 100mM sodium bicarbonate buffer for 4 hours at room temperature. Labeled Ab was separated from unconjugated label on a Zeba desalting column. Indirect immunofluorescence was performed with goat anti-mouse IgG (heavy and light chains) Alexa-Fluor 488 conjugate at 1.25-10µg/ml (Invitrogen). The UCHL1-ricin A chain immunotoxin was a gift from the laboratory of Dr. Ellen Vitetta and was made under GLP conditions as previously described [13].

**Table 1:** Antibodies used in these studies.

	**Clone**	**Fluor**	**Source**
CD45RO	UCHL1	unconjugated	Hybridoma
		FITC, PE, PerCP-Cy5.5,APC	eBioscience, BD, Icyt
		biotin	described in methods
CD45RA	5H9	FITC	BD
CD3	SP34-2	FITC,PerCP, APC-Cy7	BD
CD4	CD4R1, L200, S3.5	Alexa488, PerCP, Qdot605	NIH, BD, Life Technologies
CD8	RPA-T8	Pacific Blue	BD
CD95	DX2	APC	eBioscience
CD28	CD28.2	PerCP-Cy5.5	eBioscience
CCR5	3A9	PE	BD
CCR7	3D12	APC	eBioscience
CD2	S5.2	FITC	BD
CD20	L27	PerCP	BD
CD14	M5E2	APC	BD
Mouse IgG	Polyclonal	Alexa488	Invitrogen
Ricin A chain	RAC18	Alexa488	[40]

### Cell preparations

Flow cytometry was performed on either fresh whole blood, freshly isolated cells from blood and tissues, or cryopreserved preparations of isolated cells. Pigtailed and rhesus macaques used in these studies were either infected with SHIV-Env (162.P4) or were uninfected. The infected macaques had been aviremic for >6 months, and had normal CD4 counts. We have found no differences in CD45RO expression between uninfected, and SHIV-infected macaques, and have used the samples interchangeably; their infection status is indicated in the appropriate figure captions. To isolate peripheral blood mononuclear cells (PBMC), 10-20ml fresh blood was diluted 1:1 RPMI 1640 medium (Gibco) and layered over lymphocyte separation medium (MP Biomedicals) in a 50ml conical tube at room temperature. Tubes were spun for 30 minutes at 400xg without brake in a swinging bucket centrifuge. The buffy coat was harvested and washed in RPMI. Lymphoid tissues were teased apart in RPMI medium at 4^0^, passed through nylon filters, and washed twice in cold RPMI. Spleens and other preparations contaminated with erythrocytes were lysed in ammonium chloride lysis buffer (Pharma Lyse, BD Biosciences) for 5 min at room temperature and washed. Intestinal lymphocytes were isolated following protocols described elsewhere [22,23]. Briefly, tissue samples comprising 10 longitudinal cm of gut were cut into 0.5-1 cm^2^ sections, and washed extensively with Hank’s balanced salt solution (HBSS) at 4^0^. Samples were then placed in 50 ml conical tubes with HBSS + 10% fetal bovine serum (FBS, Hyclone Labs) + 0.75mM EDTA (Sigma) at 37^0^ with orbital shaking for 30 min. The medium was removed, the intraepithelial lymphocytes spun, resuspended in cold RPMI medium and placed on ice. Fresh warmed HBSS/EDTA was placed on the tissue pieces, and this process repeated two additional times. The tissue samples were then finely minced and placed into RPMI 1640 + 10% FBS + 60U/ml type II collagenase (Sigma) and incubated at 37^0^ with shaking. Every 30 min the tissues were further disrupted by vigorous pipetting, the supernatant removed and passed through a 40 mesh filter. The lamina propria lymphocytes were washed and resuspended in cold RPMI. Fresh warmed RPMI and collagenase were added to the tissues. The process was repeated three times. The two sets of lymphocytes were pooled and further purified by passage over very loosely-packed glass wool columns, and a gradient of lymphocyte separation medium. Viability of isolated cells was assessed by trypan blue dye exclusion. Isolated cells were frozen in 1ml aliquots at 5-20 X 10^6^ cells/ml. Cryopreservation medium containing 10% DMSO (Sigma) was used and cells were frozen using a controlled rate freezer before transfer to liquid nitrogen.

### Cell Culture

Cryopreserved cells were removed from liquid nitrogen and immediately thawed in a 37^0^ water bath. Cells were added to a 50ml conical tube containing 1ml of PBS/Bovine Pancreas DNAse I 500u/ml (Sigma). Over 4 minutes, 20ml of PBS/5% FBS/DNAse I (12.5u/ml) was added to the tube. Cells were centrifuged 300xg at room temperature for 15 min and resuspended in RPMI 1640 with L-glutamine 2mM, Penicillin G 100µg/ml, gentamicin 10µg/ml, 10% FBS, insulin 8µg/ml, oxaloacetic acid 1mM, and sodium pyruvate 500µM (all from Sigma). Prior to staining, cryopreserved cells were incubated at 2 X 10^6^ cells/ml in sterile six well plates at 37^0^, 5% CO_2_ for 4 hours. To establish long-term primary T-cell cultures, freshly explanted or cryopreserved PBMC were incubated at 2 X 10^6^ cells/ml in tissue culture medium with 10µg/ml of PHA-M (Sigma). After 2 days, the medium was replaced with fresh medium without PHA, and 24 hr later 200U/ml human IL-2 (AIDS Research and Reference Reagent Program) was added. Every 3-4 days thereafter, the medium was replaced with fresh medium + IL-2. Cells were split as necessary to prevent overgrowth. Prior to use in flow cytometry, cells were counted, washed, and resuspended in PBS at 10^6^ cells/ml.

### Flow cytometry

Flow cytometry was performed at three different sites, each using slightly different protocols and flow cytometers. Freshly explanted cells from pigtailed macaques were studied at WaNPRC. Cells (10^6^) were suspended in RPMI, 2% FBS, 0.02% sodium azide and stained in 12 X 75mm plastic tubes in separate 3-4 color panels (FITC, PE, APC, and PerCP) using reagents from BD Biosciences for 30 minutes, washed in PBS, and fixed in 1% paraformaldehyde. At least 10^5^ cells were analyzed on a FACS Calibur cytometer (BD Biosciences), compensation was performed with single stained controls. At Advanced Biosciences Laboratories, freshly explanted or cryopreserved rhesus macaque cells were stained in a similar manner. Cells were stained in 4-7 color panels using reagents purchased from iCyt. UCHL1 mAb was conjugated to FITC, PE, APC, or PerCP-Cy5.5. Live and dead cells were discriminated with Aqua Blue Live/Dead stain (Invitrogen). At least 10^5^ cells were analyzed on a LSR-II cytometer (BD Biosciences), compensation was performed with single stained controls. At the Research Institute for Children, in New Orleans, cells from humans and macaques were studied. Live and cryopreserved human PBMC were analyzed. Fresh macaque cells from TNPRC were also studied, as were cryopreserved cells from TNPRC and WaNPRC. Cells were added to each well of a 96-well plate, were pelleted in the plate for 5 minutes at 500xg, and resuspended in 50µL of cold PBA (PBS, 1% bovine serum albumin, 0.01% sodium azide) with the indicated Abs. Abs for direct immunofluorescence were incubated with cells for 30 minutes at 4^0^ in the dark before two washes with PBA and overnight fixation with 2% paraformaldehyde (Sigma Chemical). Primary Abs for indirect immunofluorescence were incubated with cells in PBA for 30 min at 4^0^ in the dark, followed by two washes and incubation with the secondary Ab (1.25-10 µg/ml) under the same conditions. Cells were then fixed. Cryopreserved lymphocytes were stained with Live/Dead near-IR stain (Invitrogen) for 30 minutes in PBS in the dark before mAb staining. Data from cells were acquired on the LSR-II flow cytometer (BD), a minimum of 10^4^ cells (for homogeneous populations) or 10^5^ cells (mixed populations) were studied. Data analysis was performed with FlowJo software (Treestar). Cells were first gated for singlets (FSC-H vs FSC-A), then for lymphocytes using SSC-A vs FSC-A (unless stated otherwise), and then for live cells when a live/dead stain was included. Controls at all sites included single stains (for compensation), and fluorescence minus one (FMO) corresponding to the UCHL1-fluorophore for gating UCHL1+ cells.

### In vivo treatment with anti-CD45RO immunotoxin or mAb

Three aviremic SHIV-infected macaques were treated with UCHL1-ricin A chain immunotoxin. 50µg/kg was administered intravenously, followed by 150µg/kg on day 3 and 450µg/kg on day 7. Three different macaques were treated with purified, sterile, low endotoxin (<0.125 EU/mg), IgG preparation of UCHL1. Fifteen mg/kg was administered intravenously over 1 hr followed three days later by 50mg/kg. No adverse events were observed. Animals were bled serially, every 3-4 days for 2 weeks, and weekly thereafter. Cells were stained for cell surface antigens Complete blood counts and blood chemistry were performed, as well as PBMC and plasma collection, and viral load monitoring. Samples were obtained prior to and 1hr post administration of the third dose of immunotoxin and the first dose of mAb, and stained to detect cell-bound immunotoxin or Ab.

### ELISA for anti-UCHL1 Ab

Anti-UCHL1 antibody was detected in macaque plasma by ELISA [24]. Briefly, ELISA plates were coated with 1µg/ml UCHL1 overnight at 4^°^C before blocking in blotto buffer. Macaque plasma was diluted in blotto buffer to 1:1000, plated in triplicate in 100µl/well, the plates incubated overnight at 4^°^C, washed 6x, and 100µl goat anti-mouse IgG-alkaline phosphatase (Invitrogen) was added at 2µg/ml for 4 hours at room temperature. Plates were washed 6 and *p*-nitrophenyl phosphate substrate (Sigma) 0.5 mg/ml in 9.8% diethanolamine was added. Optical absorbance at 405nm was read at 7 minutes on an EL320 plate reader (Bio-Tek).

## Results

### UCHL1 detects CD45RO on PBMC from all pigtailed and some rhesus macaques

**Figure 1:**
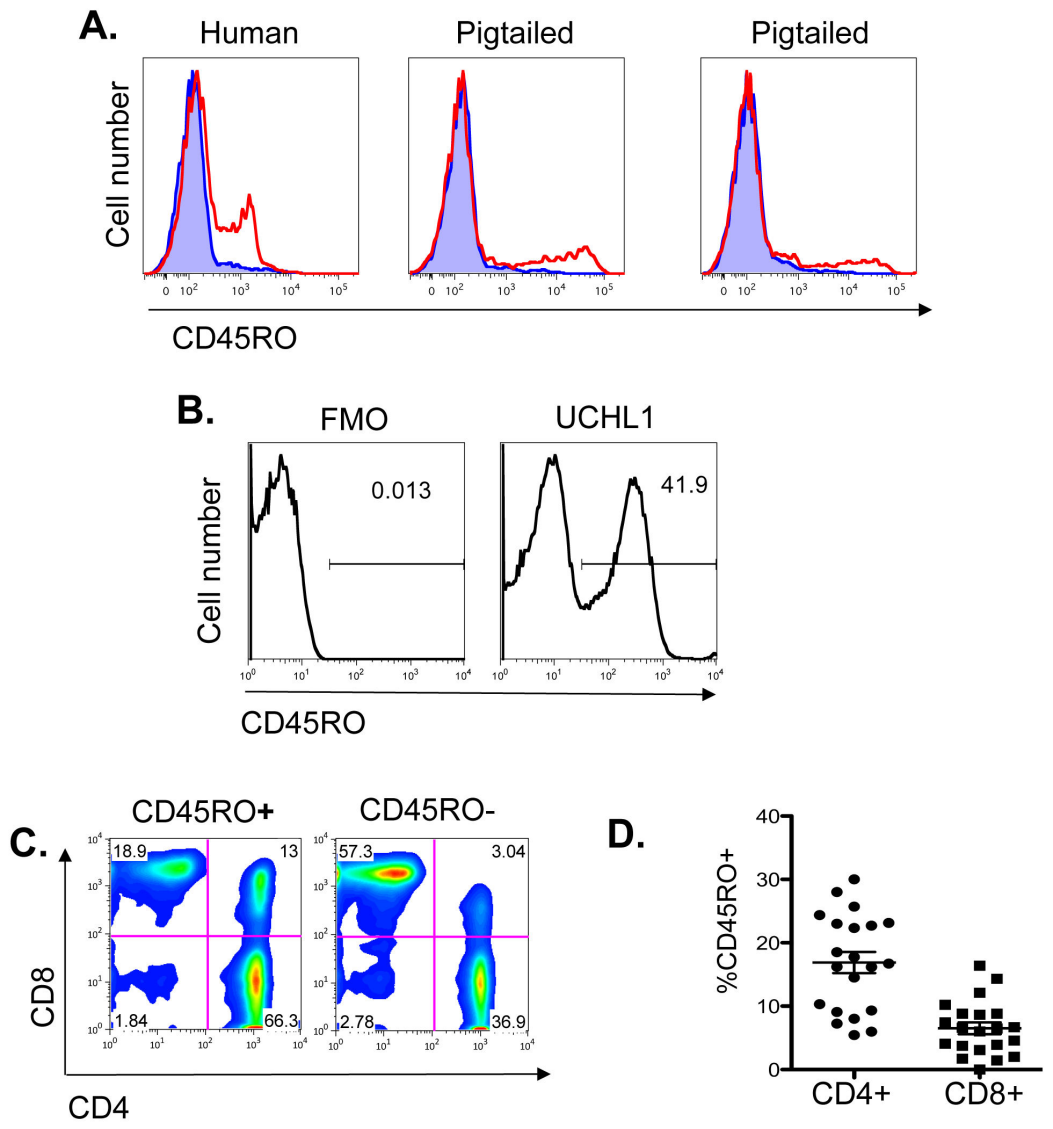
Expression of CD45RO on PBMC of pigtailed macaques. Flow cytometry was performed on freshly isolated PBMC. **A**. CD45RO was detected by indirect immunofluorescence on PBMC from two uninfected pigtailed macaques and for comparison, a human sample. Cells were gated on singlets and lymphocytes by light scatter. Murine IgG2a isotype controls are shown in blue. Data represent 1 of 3 independent experiments. **B**–**D**. Direct immunofluorescent staining of PBMC with a panel of Abs to CD3, CD4, CD8, and CD45RO. Cells were gated on lymphocytes by light scatter, then on CD3+. Panel B shows fluorescence minus one (FMO) control used to determine the CD45RO gate (left) and UCHL1 stained sample (right) in a SHIV-infected macaque (representative of 2 independent experiments). In panel C, we examined CD4 and CD8 on T-cells gated for CD45RO expression (one of 21 macaques so tested). The proportion of CD4 or CD8 PBMCs expressing CD45RO in 21 SHIV-infected macaques is shown in panel D.

Because earlier studies indicated that CD45RO might be expressed in pigtailed macaques, we first examined the binding of UCHL1 to their PBMC. In Figure 1A we show binding by indirect immunofluorescence and flow cytometry. CD45RO was detected on both human and macaque lymphocytes, but optimal staining required a higher concentration of UCHL1 on macaque cells (10µg/ml) than human PBMC (45ng/ml). We then used direct immunofluorescence with PE-conjugated UCHL1 (Figure 1B–D) in a panel with labeled Abs to CD3, CD4, and CD8. Figure 1B shows the FMO control lacking UCHL1-PE (left), which we used to draw our gates, and UCHL1-PE binding to T-cells (right). Figure 1C shows in a single macaque that CD45RO+ cells are predominantly CD4+, whereas CD45RO- are more often CD8+. The proportion of CD4+ and CD8+ cells expressing CD45RO in 21 macaques is shown in Figure 1D. These results indicate that CD45RO is more often found on CD4+ than CD8+ lymphocytes in pigtailed macaques, and that this marker could be detected in all animals of this species. We also examined CD45RO expression on monocytes, PMNs, B and NK cells, and found it to be lacking (not shown).

**Figure 2:**
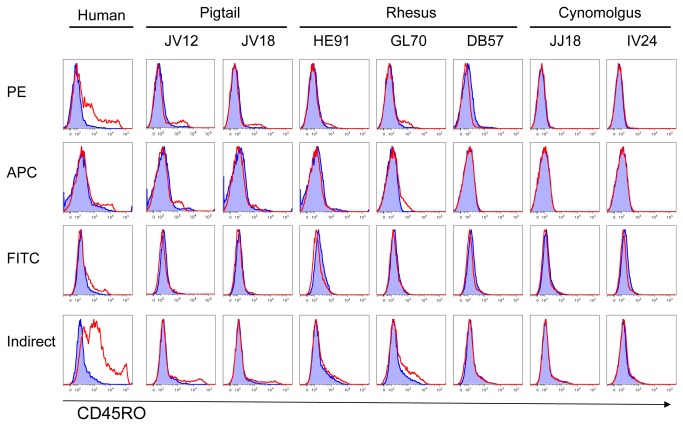
Binding of UCHL1 to lymphocytes from different macaque species. Cryopreserved PBMC from human, and uninfected pigtailed, rhesus and cynomolgus macaques were stained with commercially prepared UCHL1 conjugates with PE, APC, or FITC according to the manufacturer’s recommendation, or by indirect immunofluorescence using 10 µg/ml of unconjugated UCHL1 (red histograms). Directly conjugated isotype controls were run for human and pigtailed macaques (blue histograms), and unstained controls for rhesus and cynomolgus. Data representative of n=2 independent replicate experiments.

**Figure 3:**
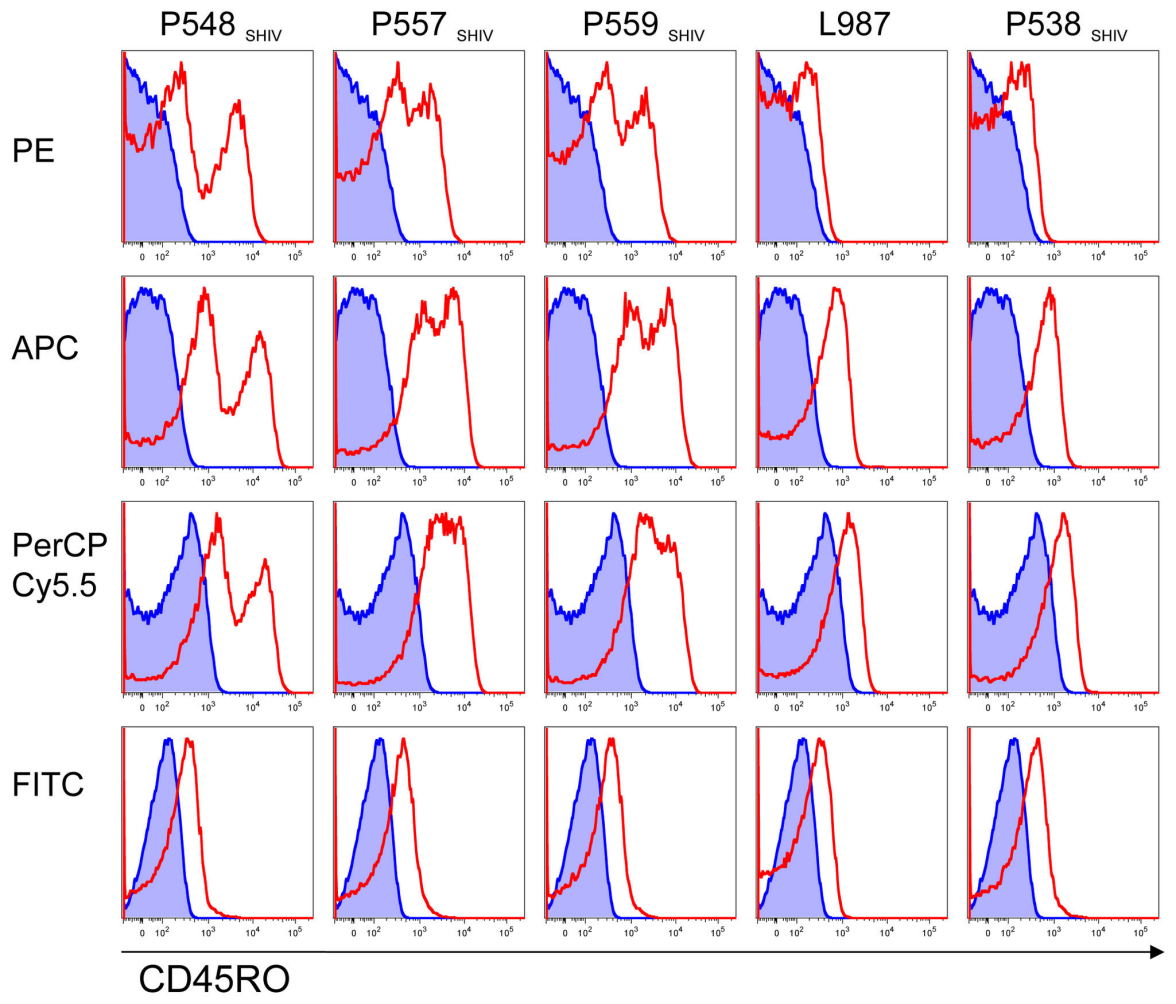
Binding of directly conjugated UCHL1 to lymphocytes from rhesus macaques. Direct immunofluorescence was performed on freshly isolated PBMC from four SHIV-infected and one uninfected rhesus macaques. UCHL1 conjugated to PE, APC, PerCP Cy5.5, or FITC and Ab to CD3, CD4 and CD8 were used. Fluors used in each panel were adjusted to accommodate the fluor on UCHL1. Cells were gated on singlets and lymphocytes by light scatter, and then on CD3+ CD4+ cells. FMO controls are shown in blue.

In Figure 2 we examined CD45RO expression on lymphocytes from macaques of other species, since rhesus and cynomolgus macaques are widely used in research. We also compared the performance of conjugates made with different fluorochromes and unconjugated UCHL1 measured by indirect immunofluorescence. CD45RO was detected on lymphocytes from human and both pigtailed macaques but only in some rhesus macaques. No reactivity was found in the two cynomolgus macaques tested. Indirect immunofluorescence and direct immunofluorescence with PE and APC conjugated UCHL1 detected similar frequencies of CD45RO+ cells in macaques, whereas the FITC conjugate detected none. The presence of CD45RO on lymphocytes from some rhesus macaques prompted us to investigate further. Expression of CD45RO on CD4 T-cells from PBMC from an additional five rhesus macaques was examined (Figure 3). Three of the five macaques expressed CD45RO on CD4 T-cells as detected by the PE, APC and PerCP Cy5.5 UCHL1 conjugates. As before, the FITC conjugate did not react. The shift in fluorescence of the negative peaks from the FMO control suggests either a small degree of nonspecific sticking to cells by UCHL1, or perhaps the presence of a cross-reacting carbohydrate epitope found on many more cell types.

**Figure 4:**
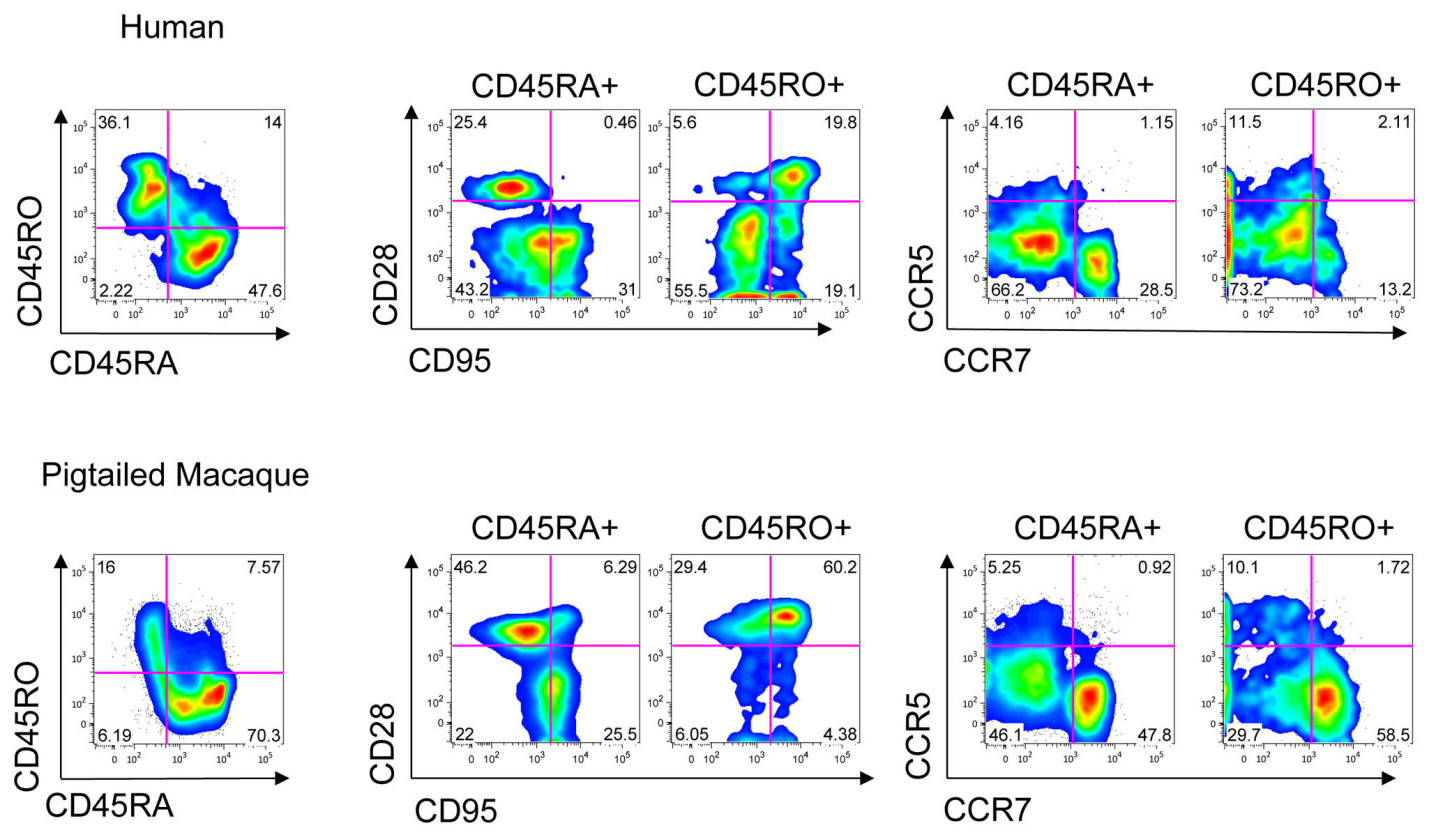
Memory markers on PBMC. Cryopreserved PBMC from an uninfected pigtailed macaque and a human were stained with panels of Abs to memory markers (CD45RO, CD45RA, CD95, CD28 or CD45RO, CD45RA, CCR7, CCR5). Cells were gated on singlets and lymphocytes by light scatter, and then gated on single-positive CD45RO or CD45RA. Human cells are displayed in the top row and macaque cells in the bottom row. Left: CD45RO and CD45RA expression on lymphocytes, showing gates used to distinguish CD45RO+ and CD45RA+ cells in center and right panels. Center: CD95 and CD28 expression on CD45RO and CD45RA single-positive cells. Right: CCR7 and CCR5 expression CD45RO and CD45RA single-positive cells. Data are from a single experiment.

CD45RO+ cells from a pigtailed macaque were studied for other markers characteristic of memory T-cells (Figure 4). Lymphocytes were tested for the expression of CD45RO, CD45RA, CD95, CD28, CCR5, and CCR7. We compared the expression of these markers in pigtailed macaques and humans. Macaque cells were predominantly CD45RA+ CD45RO-, with a smaller proportion of single-positive CD45RO+ and double-positive CD45RO+ CD45RA+ cells. Human cells had a higher proportion of CD45RO+ cells. In macaque, CD95 expression was greater in the CD45RO+ population than the CD45RA+ population. High CD28 expression in the CD45RO+ population suggests these lymphocytes were of a central or transitional memory phenotype. A difference between human and macaque CD45RO+ cells is the presence in the human of a large CD28-CD95- population that is lacking in the macaque. In the macaque there is a small population of CD45RA+ CD95+ CD28+ cells that is absent in the human. In macaque, expression of CCR5 (tissue homing [25,26]) and CCR7 (secondary lymphoid tissue homing [27]) were similar in the CD45RO+ and CD45RA+ populations. CD45RO+ cells in human had lower expression of CCR7.

### UCHL1 binds to macaque CD45RO less avidly than to human

**Figure 5:**
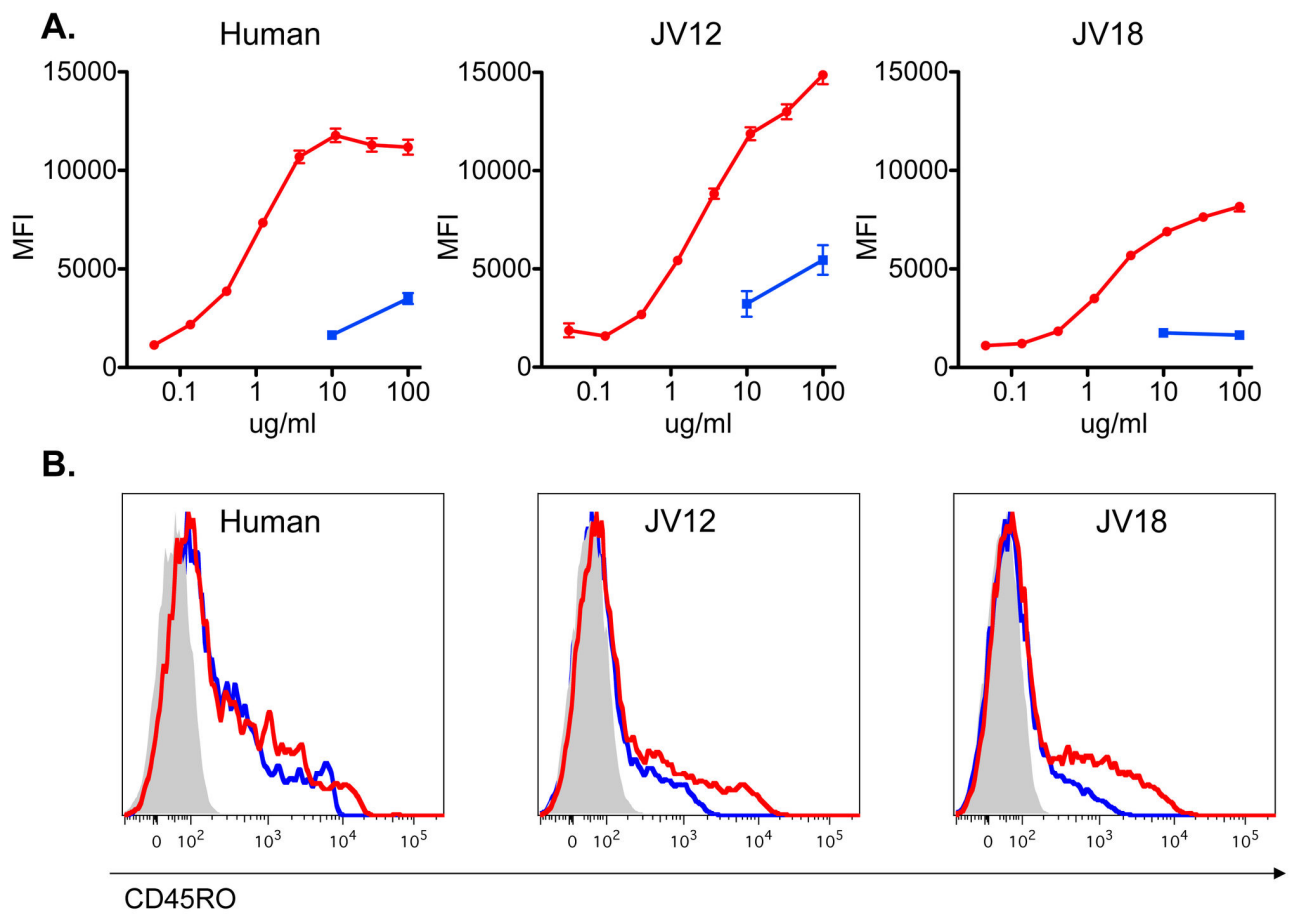
Relative avidity of UCHL1 for human and pigtailed macaque CD45RO. **A**. Indirect immunofluorescence and flow cytometry were performed on human and 2 sets of pigtailed macaque (JV12 and JV18) PBMC. Equal numbers of thawed, rested PBMC were incubated with serial dilutions of UCHL1 before staining with an excess of Alexa-488 conjugated anti-mouse IgG. Acquired cells were gated on singlets and lymphocytes by light scatter. A gate for UCHL1 positive cells was then drawn. Data presented in red are the mean fluorescent intensity of the positive cells. Staining of cells by IgG2a isotype controls are shown in blue. Data representative of n=3 experiments. **B**. Human and pigtailed macaque (JV12 and JV18) PBMC were stained with biotinylated UCHL1 and streptavidin-PE in a direct or indirect staining method. For the direct method, biotinylated mAb was incubated with streptavidin-PE for twenty minutes before adding the multimeric complex (to a final concentration of 20µg/ml Ab and 2µg/ml streptavidin-PE) to the cells. For the indirect method, cells were first incubated with 20µg/ml of biotinylated mAb for thirty minutes, washed thoroughly and then incubated with 2µg/ml of streptavidin-PE. Acquired cells were gated on singlets and lymphocytes by light scatter. Direct immunofluorescence with multimeric UCHL1 is shown in red. Indirect immunofluorescence with monomeric UCHL1 is shown in blue. Autofluorescence of unstained PBMC is shown in grey. Data representative of a single experiment.

We had previously observed that compared to human, pigtailed macaque PBMC required a higher concentration of UCHL1 for optimal staining (Figure 1). This suggested that UCHL1 may have lower avidity for macaque CD45RO than for human. An alternative explanation is that although macaque and human CD45RO have identical structures, but there is less of it on the surface of macaque cells than human. In Figure 5A, we performed indirect immunofluorescence with serial dilutions of UCHL1 on equal numbers of human and pigtailed PBMC. The mean fluorescent intensity of CD45RO+ lymphocytes reached saturation between 3–10µg/ml for human cells, whereas the macaque cells had not reached saturation at 100µg/ml. This is consistent with the hypothesis that UCHL1 has lower avidity for macaque CD45RO than human CD45RO. The maximum heights of the curves of JV12 and JV18 compared to human may indicate a higher or lower concentration of antigen on the surface of these cells. We next sought to improve the avidity of UCHL1 for macaque CD45RO by increasing the valency of the mAb. In Figure 5B, we biotinylated UCHL1 and incubated the mAb with streptavidin-PE. Up to four mAbs may bind the tetravalent streptavidin, creating a multimeric Ab complex. The resulting multimer was used in direct immunofluorescence to stain human and pigtailed macaque PBMC. For comparison, indirect immunofluorescence was performed in parallel. Cells were incubated with monomeric biotinylated mAb, washed, and then streptavidin-PE was added for detection. Improved binding by the multimeric complex compared to monomeric Ab was observed on macaque cells, but not human. These data support the hypothesis that UCHl1 binds to a high prevalence low-avidity cross-reactive epitope, and that the decreased avidity of monomeric UCHL1 can be improved by making multimeric UCHL1 complexes, although we have not ruled out the possibility that differences in epitope density also play a role.

### CD45RO expression in tissues

**Figure 6:**
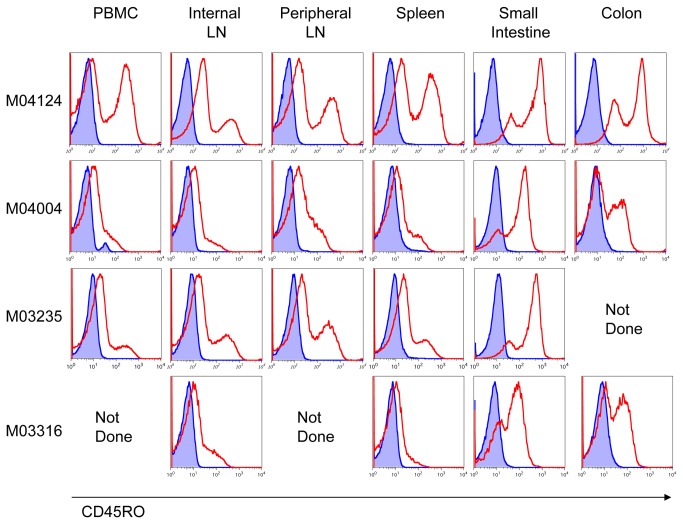
CD45RO on T-cells isolated from tissue. Lymphocytes were isolated from tissue explants taken at the time of necropsy from one uninfected (M04004) and three aviremic SHIV-infected pigtailed macaques. Lymphocytes were pooled from the mesenteric and bronchial lymph nodes (internal LN) and from the axillary and inguinal lymph nodes (peripheral LN). Cells were then stained with Abs to CD3, CD4, CD8, and CD45RO and analyzed by flow cytometry. Acquired cells were gated on lymphocytes by light scatter, and then on CD3+ cells. Stained cells are shown in red. Unstained controls are shown in blue. Data representative of n=5-6 macaques.

**Figure 7:**
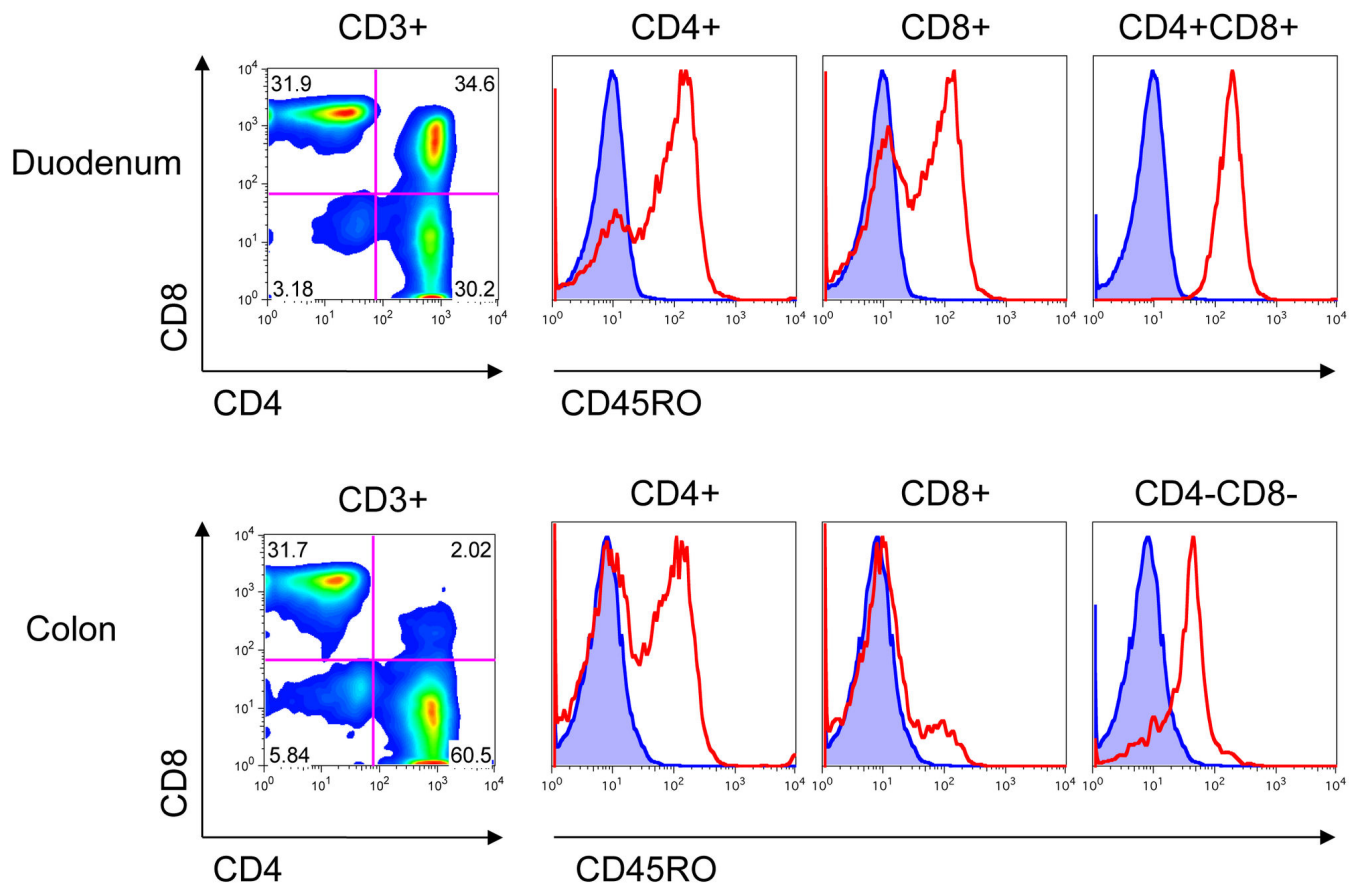
CD45RO expression on T-cells in the gut. Lymphocytes were isolated from GALT of colon and duodenum of a SHIV-infected pigtailed macaque at time of necropsy and stained with Abs to CD3, CD4, CD8, and CD45RO. Cells were analyzed by FACS. Lymphocytes were gated by light scatter, and then on CD3+ cells. Cells were further gated on CD4 and CD8. Expression of CD45RO is shown in red. Unstained cells are shown in blue. Data representative of n=5 macaques.

The detection of CD45RO on PBMC in pigtailed macaques encouraged us to examine the expression of CD45RO in other anatomical locations. In Figure 6 lymphocytes isolated from the tissues of one uninfected and three aviremic SHIV-infected macaques were stained with a panel of Abs to CD3, CD4, CD8 and CD45RO. In addition to the PBMC, CD45RO+ T-cells were found in the internal lymph nodes, peripheral lymph nodes, spleen, and the gut associated lymphatic tissue (GALT) of the small intestine and the colon in each macaque. The highest expression of CD45RO was in the gut. CD45RO+ cells were also present in the thymus (data not shown). As observed earlier, it appears that for CD45RO- cells there is a degree of UCHL1 binding that is greater than background. This varies by animal and by tissue. We examined T-cells in the gut that were double-positive CD4+ CD8+ or double-negative CD4-CD8- and found that they too expressed CD45RO (Figure 7). All of the CD4+ CD8+ T-cells in the duodenum were CD45RO+.

**Figure 8:**
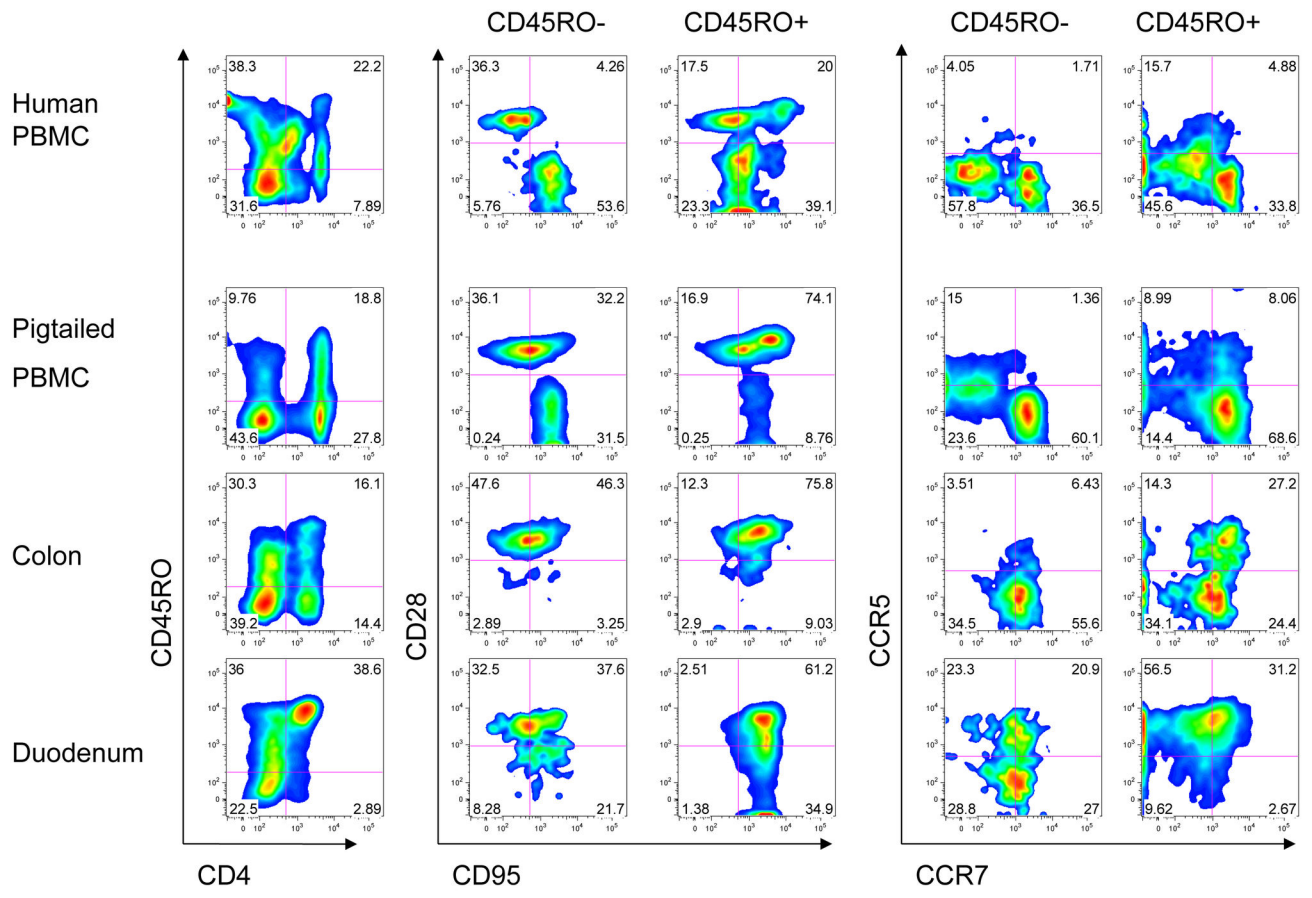
Expression of memory markers on CD4+ lymphocytes from different tissues. Cryopreserved lymphocytes from GALT of colon and duodenum, and PBMC from an uninfected pigtailed macaque and a human subject were stained with panels of Abs (either CD4, CD45RO, CD95, CD28 or CD4, CD45RO, CCR7, CCR5). Lymphocytes from the colon and duodenum were also live/dead stained. Acquired cells were gated on singlets, live cells and then lymphocytes before gating on CD4 and CD45RO. CD45RO+ and CD45RO- subsets of CD4+ cells were analyzed for CD95 and CD28, or CCR7 and CCR5. Left: CD4 and CD45RO expression. Center: CD95 and CD28 expression on CD4+ CD45RO- and CD4+ CD45RO+ cells. Right: CCR7 and CCR5 expression of CD4+ CD45RO- and CD4+ CD45RO+ cells.

In Figure 8, CD4+ cells from GALT of the colon and duodenum, and peripheral blood were studied for the expression of memory markers: CD45RO, CD95, CD28, CCR5, and CCR7. CD4+ cells in the duodenum had a higher proportion of CD45RO+ lymphocytes than found in colon or peripheral blood. The majority of CD45RO+ cells in all tissues had a central or transitional memory phenotype (CD95+ CD28+), but in the duodenum there were also a considerable number of effector memory cells (CD95+ CD28-). In the CD45RO- population, we found cells expressing CD95 and CD28, in contrast to human. The majority of CD45RO+ cells in the duodenum expressed CCR5, whereas a smaller proportion of CD45RO+ cells in the colon and PBMC did. CD45RO- cells in the intestine expressed lower levels of CCR5 than did the CD45RO+ cells. No appreciable difference was seen in CCR7 expression in the CD45RO+ and CD45RO- populations, nor among the tissues.

### T-cell activation and CD45RO expression

**Figure 9:**
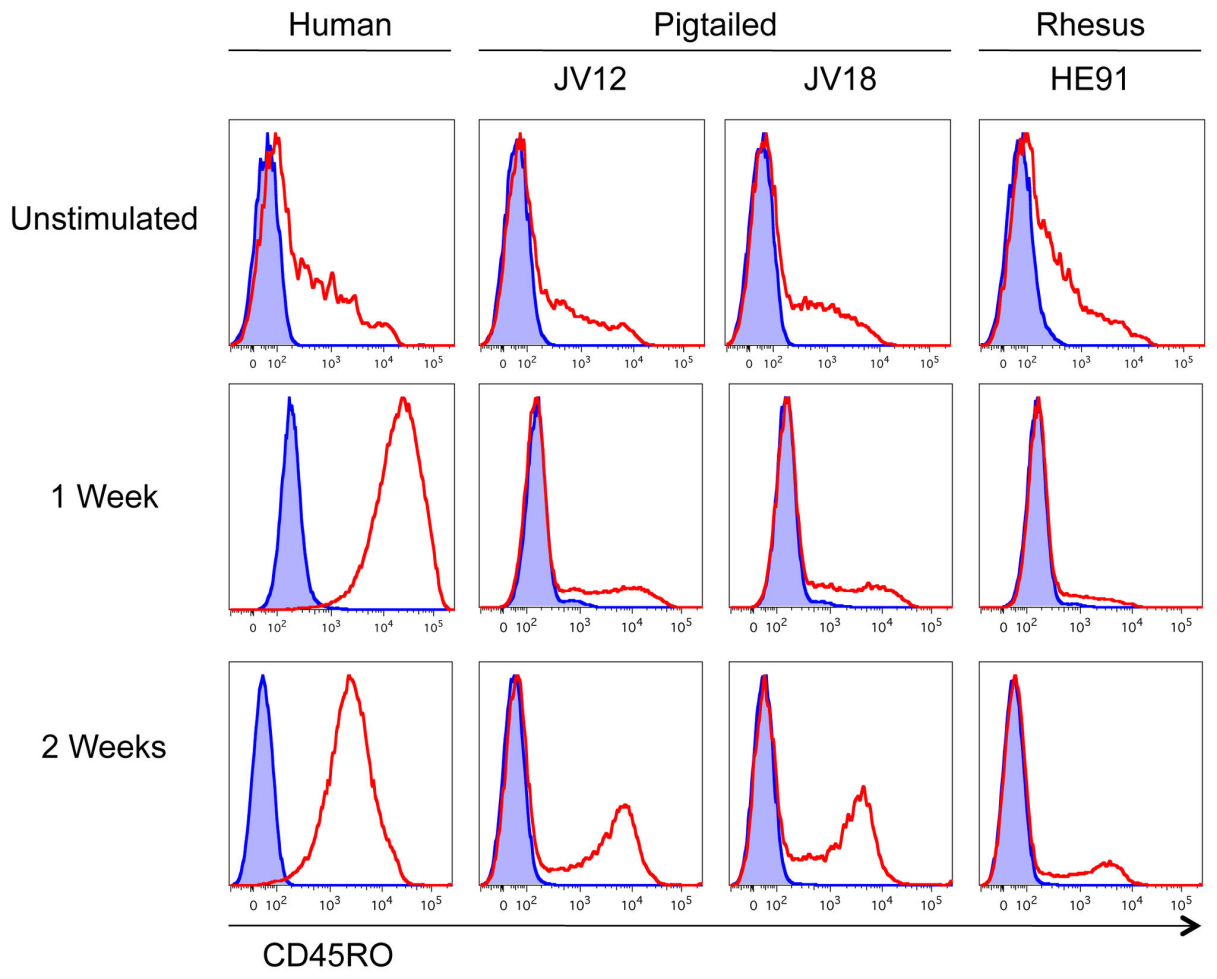
Mitogen stimulation and CD45RO expression. Cryopreserved PBMC from human, and uninfected pigtailed (JV12 and JV18) and rhesus (HE91) macaques were stained with UCHL1-multimers or were stimulated with PHA for 48 hours, and then maintained in medium containing IL-2. The resulting blast cells were stained with UCHL1-multimers on days 7 and 14. Cells were analyzed by flow cytometry. Acquired cells were gated on singlets and lymphocytes by light scatter. UCHL1-multimer staining is shown in red. Isotype controls (biotinylated IgG2a-streptavidin-PE multimers) are shown in blue.

It was previously observed that human PBMC blast cultures, only a proportion of which are initially CD45RO+, uniformly express CD45RO within one week in culture [7]. We compared expression of CD45RO in cultures of macaque and human PBMC activated with PHA and maintained in IL-2. Seven days after the initial stimulation, more than 90% of human blasts were CD45RO+ (Figure 9). In contrast, CD45RO was expressed on only a small proportion of macaque cells and this did not increase with time. In human PBMC, the intensity of CD45RO expression decreased between one and two weeks, whereas in the macaque cells the separation between the positive and negative populations increased in this interval. The uniform expression of CD45RO on PHA-activated human blasts, compared to the modest degree observed in macaques may imply a functional difference in the CD45RO+ populations of macaques and humans, and/or a difference in the population of cells that is activated by PHA.

### In vivo administration of UCHL1 mab or immunotoxin

**Figure 10:**
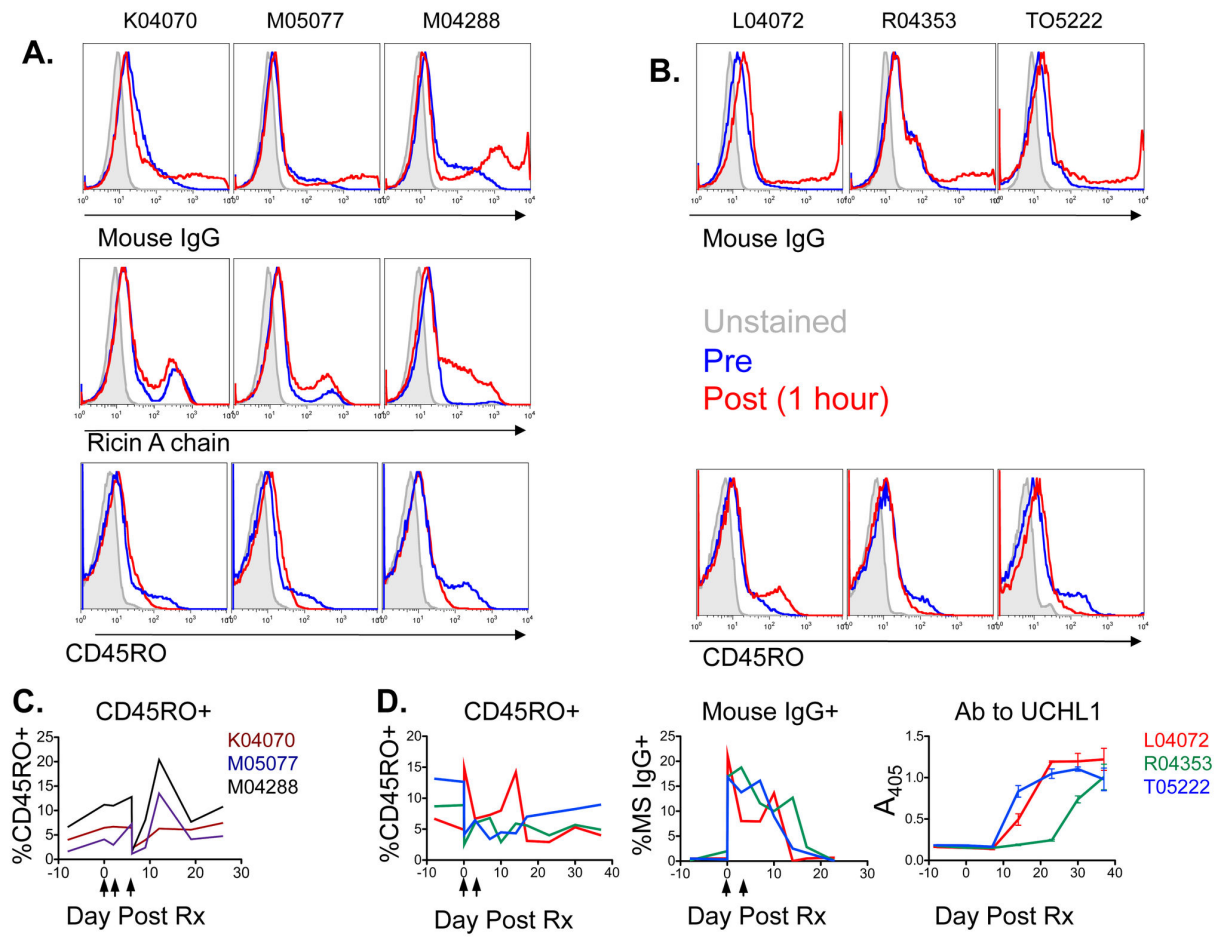
In vivo administration of UCHL1 immunotoxin or mAb. Groups of three aviremic SHIV-infected macaques each received escalating doses of UCHL1-ricin A chain immunotoxin (panels A and C) or UCHL1 mAb (panels B and D). **A**. Flow cytometry was performed prior to (blue histograms) and 1hr following (red) administration of the third dose of UCHL1-ricin A chain immunotoxin. Cells were stained with antibody specific for mouse IgG (top), ricin A chain (middle), or CD45RO (bottom). **B**. Flow cytometry was performed prior to and one hour following the first administration of UCHL1 mAb. Cells were stained with antibody specific for mouse IgG (top) or CD45RO (bottom). **C**. Percent of CD3+ cells that express CD45RO was followed with time using flow cytometry. Day 0 indicates the start of immunotoxin administration (vertical arrows). **D**. The effects of administering UCHL1 mAb were followed over time. Day 0 indicates the start of mAb administration (vertical arrows). Left: percent of CD3+ cells that express CD45RO, measured by flow cytometry. Middle: percent of cells detected with fluorescent anti-mouse IgG, measured by flow cytometry. Right: macaque anti-UCHL1 antibody measured by ELISA.

To determine if UCHL1 Ab would bind to cells when administered in vivo, groups of three pigtailed macaques received either an immunotoxin consisting of UCHL1 conjugated to ricin A chain (total dose 650µg/kg in 3 divided doses), or unconjugated UCHL1 (total dose 65 mg/kg in 2 divided doses). The results are shown in Figure 10. Figure 10A shows samples obtained immediately before and 1hr following the third dose of UCHL1-ricin A chain. There is a marked increase in both the number of cells with mouse IgG, and the quantity of IgG on the positive cells. The same is true for ricin A chain on the cell. Conversely, the number of CD45RO+ cells declined. Figure 10B shows similar results in macaques treated with unconjugated UCHL1. However, one macaque, L04072, showed an increase in the number of CD45RO+ cells. Since this is the only macaque of 6 tested that showed such an increase, we believe this result is most likely laboratory error. Figure 10C shows that the proportion of CD45RO+ cells in the circulation of immunotoxin treated animals drops in the first days following treatment, but rapidly returns to normal. In Figure 10D, a less pronounced drop in the proportion of CD45RO+ cells is observed following mAb treatment. Robust depletion of CD45RO+ cells in the peripheral blood was not observed, and numbers of CD45RO+ cells quickly returned to pretreatment values. Figure 10D also shows that circulating cells carrying mouse IgG persist for 2-3 weeks, before they rapidly disappear. The inverse relationship between CD45RO+ cells and msIgG+ cells suggests that the apparent loss of CD45RO+ cells is best explained by blocking of the binding of the fluorescent detecting Ab, by the administered Ab. At the same time that msIgG+ cells decrease, the animals develop an Ab response to UCHL1 (Figure 10D). The timing of these events suggests that the macaque anti-mouse Ig response leads to the clearance of circulating UCHL1. 

## Discussion

We have shown that CD45RO is expressed on a subset of T-cells in all pigtailed and some rhesus macaques (Figures 1-3). CD45RO was not expressed on lymphocytes from the few cynomolgus macaques we examined. These results are consistent with those of Firpo et al, who found UCHL1 was cross-reactive with pigtailed and rhesus macaques by indirect immunofluorescence [17]. In the recent study by Wang [18], CD45RO (as identified by the mAb OPD4) was expressed by nearly all of the pigtailed macaques, many of the Indian-origin rhesus macaques, and none of the Chinese-origin rhesus macaques. Thus, the proportion of individual macaques by species which react with either UCHL1 or OPD4 is similar. These data and ours suggest that mAbs against CD45RO can be useful reagents to identify, deplete or monitor memory cells in pigtailed macaques, whereas rhesus macaques will require an initial screening for reactivity. Because OPD4 is not readily available in the U.S., the demonstration that UCHL1 does react with macaque memory cells is of great utility and provides an immunological tool for macaque models.

Our results explain how confusion arose regarding expression in macaques of CD45RO, as defined by UCHL1. The cross-reaction of UCHL1 with the macaque epitope is of lower avidity than binding to human CD45RO, the immunogen. This weaker binding may be more easily disrupted by chemical modifications within the mAb’s CDRs during conjugation to a fluor. In biotinylating UCHL1, we found it particularly sensitive to effects of conjugation: labeling at 1: 1 molar ratio of biotin to mAb retained binding activity (Figure 5), but with a five-fold molar excess of biotin, binding was partly inhibited, and a ten-fold excess of biotin completely eliminated binding to CD45RO (data not shown). Analysis of the hypervariable regions of UCHL1 (Genbank Accession KC295246, KC295247) found that there are two lysine residues and 24 hydoxylated residues (serine or threonine) within the CDRs which could potentially react with the NHS-esters commonly used to label mAbs [28]. FITC has a lower quantum efficiency than newer dyes, and thus FITC is frequently conjugated at higher fluor: Ab ratios. As shown in Figures 2 and 3, FITC conjugates of UCHL1 (from 2 different manufacturers) fail to cross-react with macaque cells. Because FITC is the least expensive fluorochrome, it is commonly used. The lack of binding with a FITC-conjugated UCHL1 from a well-regarded commercial source would normally indicate that the antigen is not present.

The structure of the epitope bound by anti-CD45RO mAbs has not been precisely defined. Previous studies indicate that UCHL1 recognizes a carbohydrate or carbohydrate dependent epitope that is formed from the juxtaposition of three O-linked glycans when exons 3 and 7 are spliced in the CD45RO isoform [8]. In addition to the expected reactivity, UCHL1 demonstrated a low level of binding to many cells (Figures 2, 3 and 6). Both the degree of binding and the proportion of cells showing such binding varied from animal to animal, and in different tissues. Some of the variability may be due to experimental protocols. For example Figure 2 uses cryopreserved cells. Figure 6 uses fresh. It has previously been reported that there is a “consistent decrease in the expression of CD45RO molecules” upon cryopreservation [29]. Isotype controls are capable of nonspecific binding, which might change the background to which UCHL1 binding is compared [30]. Multicolor staining could increase spillover into the channel of interest. This would be best controlled by the use of a “fluorescence minus one” control. While such controls were not used in this particular experiment, they were included in Figure 3, where a large shift of the CD45RO- peak is observed, when compared to the FMO control. But we also believe that there are real biological differences in the degree of low level UCHL1 binding that are based on anatomic milieu and inter-animal variation

We have observed low level binding of UCHL1 to lymphocytes that would be considered CD45RO-. This “background” binding varies in intensity in cells of different animals, and on cells from different tissues, but is always distinguishable from UCHL1’s binding to CD45RO+ cells. We cannot determine whether UCHL1 binds to a high prevalence cross-reactive glycan with low affinity because of structural differences in the glycans, or whether there are very small amounts of the same (i.e. CD45RO) glycan on the surface of these cells. To define the epitope in greater detail, we tested the binding of UCHL1 to glycan and glycopeptide arrays [31,32], but none of the interactions reached a level of significance (Ola Blixt, data not shown). Currently, we can only speculate as to the nature of the difference in the structure of the UCHL1 epitope in humans and macaques. However, these results do indicate that the difference between CD45RO+ and CD45RO- is a matter of degree, rather than absolute expression. This is common among carbohydrate antigens expressed on cell surfaces. For example, we have found mAbs raised against the capsular polysaccharide of group B Streptococcus can bind glycans on human cells [33].

The expression of CD45RO in macaques is consistent with a memory phenotype and its distribution is similar, but not identical, to that in humans. CD45RO is predominantly expressed on helper T-cells in both human and macaque (Figure 1C and 1D). Macaque CD45RO+ cells have high levels of the markers CD95 and CCR5, which are enriched on memory cells, the latter particularly in gut-associated tissues (Figures 4 and 8). In macaques, memory cells have been subdivided into subsets based on the expression of CD28, CD95, and CCR7, where CD95+ CCR7**-**CD28+ defines transitional memory T cells (T_TM_), CD95+ CCR7- CD28- defines effector memory (T_EM_), and CD95+ CCR7+ CD28+ central memory (T_CM_) [34]. In the PBMC and in the gut, the majority of CD45RO+ cells express both CD28 and CD95, and thus are either T_CM_ or T_TM_. CCR7 is expressed on a large majority of CD45RO+ cells, suggesting most are T_CM_ cells. But a substantial number of CD45RO+ cells are CD95+ CD28-, indicating that at least some CD45RO+ cells are of the T_EM_ phenotype, especially in the duodenum. Additionally, there are RA+ or RO- populations that express CD95. Thus it appears that CD45RO defines a set of memory cells overlapping, but different, from those defined by CD95, CD28, and CCR7. Compared to the lymph nodes and peripheral blood, the proportion of CD45RO+ cells in macaque is enriched in the GALT, where a larger number of antigen-experienced T-cells are expected (Figure 6). As in humans, CD45RO was expressed on macaque thymocytes where it is not associated with a memory phenotype [35]. In contrast to humans, detection of CD45RO was confined to T-cells.

Activated T-cells eventually convert to a memory phenotype or undergo cell death [36,37]. We observed differences in the expression of CD45RO on human and macaque lymphocytes stimulated with PHA and cultured in the presence of IL-2. After one week, nearly all of the human blast-cells expressed CD45RO, while less than half of the macaque blast-cells did (Figure 9). This may reflect species differences in the population of cells that responds to PHA, the control of CD45RO expression following cellular activation, or the population of cells defined by CD45RO [2,7].

Memory CD4 T-cells constitute the major, if not exclusive, reservoir of provirus in HIV+ individuals on long-term antiretroviral treatment [9–11]. Abs that deplete these cells could be of great therapeutic interest as a method to deplete persistent reservoirs of HIV that does not require activation of the reservoir. Vitetta and coworkers first demonstrated that latently infected cells expressed CD45RO [13–15]. More recently, T_CM_ and T_TM_ have been implicated [38,39]. These cells express both CD95 and CD27, but these markers (Fas and TNF receptor) are problematic as therapeutic targets for subset depletion. Therefore we propose the use of the UCHL1 for this purpose. But UCHL1 binds less avidly to macaque CD45RO than to human. In Figure 1, one hundred-fold less UCHL1 was required to yield equivalent binding in human compared to macaque cells. Figure 5 shows that human CD45RO was saturated with UCHL1 between 3–10µg/ml mAb, while macaque CD45RO was not saturated even at 100µg/ml. Because of UCHL1’s low avidity for macaque CD45RO and its immunogenicity, its therapeutic utility may be limited in macaque models of HIV infection. We separately examined the ability of UCHL1-ricin A chain immunotoxin and unmodified UCHL1 to deplete cells in vivo. Robust depletion of CD45RO+ cells was not observed in either case, even though both components of the immunotoxin could be found bound to cells (Figure 10A). Unconjugated Ab could be detected on the surface of circulating PBMC for three weeks following administration (Figure 10D). Loss of cells with cell-surface UCHL1 corresponded with the development of an anti-UCHL1 Ab response (Figure 10D). We did observe transient decreases in the number of circulating UCHL1+ cells following administration of immunotoxin or mAb. We believe that this is most likely due to blocking of the binding of fluorescent UCHL-1 by the UCHL1 administered to the animal, rather than actual depletion of CD45RO+ cells. But because no other CD45RO Abs that bind a different epitope than UCHL1 are available, we cannot rule out transient depletion. Multimeric complexes of UCHL1 improved binding to macaque CD45RO but not human (Figure 5B). Thus, UCHL1’s low avidity interaction with macaque CD45RO may be overcome by increasing the valence of the Ab. We are designing double variable domain Abs to study effects of Ab valence on function [24], and are preparing a tetrameric human IgG1 version of UCHL1 that may have increased in vivo depleting activity.

In this communication, we have demonstrated that macaques express CD45RO as defined by the readily available murine mAb UCHL1. In macaques, it is expressed predominantly on helper T cells (CD4), and to a lesser degree on cytotoxic T cells (CD8). We have not found CD45RO expressed on monocytes, B cells or NK cells. CD4+, CD45RO+ cells express other memory markers. The binding of UCHL1 to macaque cells is of lower avidity than to human cells, making the Ab extremely sensitive to conjugation procedures. The failure of FITC-conjugated UCHL1 to bind macaque CD45RO obscured the expression of this marker in macaques. We are pursuing the use of UCHL1 as a therapeutic Ab to deplete cells carrying functional persistent HIV provirus. To use this Ab in macaque models, we must first overcome limitations imposed by low avidity, which we propose to accomplish by increasing Ab valence. 
